# Lectures on Clinical Medicine, Delivered at the Philadelphia Medical Institute

**Published:** 1838-06-20

**Authors:** W. W. Gerhard

**Affiliations:** Physician to the Philadelphia Hospital, &c.


### CLINICAL LECTURES.

### LECTURES ON CLINICAL MEDICINE, *delivered at the Philadelphia Medical Institute, by* W. W. GERHARD, M. D., *Physician to the Philadelphia Hospital*, &c.

APOPLEXY AND INFLAMMATION OF THE BRAIN,* (CONTINUED)—FUNCTIONAL DISEASES OF THE BRAIN.

*Friday*, June 8th.—I shall, this afternoon, again call your attention to some organic and functional diseases of the brain. It was my intention to have confined myself to the subject of functional cerebral diseases, but, owing to the termination of one of the cases of apoplexy, following acute softening of the brain, noticed in the lecture on these subjects, at the post-mortem examination of which some of you were present this morning, I am induced to recur to the topic. The impossibility of understanding the subject of organic diseases of the brain without a knowledge of their pathology, is well exemplified by the case under consideration, while, on the other hand, you have seen how exactly the phenomena after death coincided with the condition, which we were able, from the symptoms, to announce during life, and how entirely our prognosis as well as our diagnosis has been confirmed by the result.

Pathological anatomy becomes a most useful aid in the study of disease when we examine thus closely the connexion between the symptoms and lesions. The changes detected after death explain the phenomena which have occurred during life, and by giving us a clearer notion of the different periods of the disease, not only facilitate diagnosis, but are of great utility in the treatment of disease. We also acquire by this means a more thorough conviction of the harmony and regularity of the laws which govern disease. You would not have so well understood the connexion between diseases of the heart and acute rheumatism, if, a few days since, in a lecture on pathological anatomy, I had not been able to demonstrate the adhesions which closely connected the heart to the pericardium and occurred during an endo-pericarditis, complicating a most severe rheumatism, which I treated successfully in the summer of 1837. The case was not the less interesting, from the fact of the patient dying of a totally different disease, (tuberculous meningitis) in a ward not under my care; so that the evidence of the former pericarditis was presented in still stronger relief than would have happened if the patient had remained under treatment. The present case is another illustration of the regular connexion of the symptoms of disease with particular lesions, and, if you have any scepticism on the subject, may convince you that in our description of disease, we do not enter the land of imagination or fable.

This case was that of Fisher, one of the blacks alluded to in Lecture VII. He entered the hospital, a short time after having been seized with loss of consciousness and other symptoms, denoting an attack of apoplexy. The inflammation, excited by the clot of blood thrown out, induced an inflammatory softening of the structure of the brain, which seems sometimes to be a useful process, and promotes absorption of the clot; it occurs from the same cause that gives rise to inflammation, wherever a foreign substance is present in any portion of the body. The train of symptoms, announcing the existence of acute softening of the brain, we treated by cupping, purging, and a regulated diet, not using general bleeding from the enfeebled condition of the patient. Under this treatment, he was slowly getting better—he could crawl about the ward, and could articulate short sentences, when yesterday, he was seized with a fit of convulsions, as the nurse termed it, and was found by one of the resident physicians in a state of coma, with dilated pupils, &c., which soon terminated in death. The examination after death, this morning, explained the occurrence of these symptoms.

We found, first, the remains of the old apoplexy, which had taken place a year ago, probably a few day's before the man's admission, as nearly as we could gather from his imperfect account of himself. The disease had occurred at several different points of the left hemisphere of the brain. The left corpus striatum was shrunken and shrivelled up, and unnaturally hard and indurated. On incising it, at the depth of the eighth of an inch, a well marked cyst was found, rather more than an inch long, and half an inch broad, lined with the usual serous membrane, which was not quite complete; a part of the walls of the cyst were composed of loose cellular substance, filled with an opaque liquid. This serous membrane, lining the cavity, was not a true but an adventitious serous membrane, or rather sero-cellular, such as is thrown out in inflammation of the pleuræ and pericardium.

I have already told you in a previous lecture, that these cysts are left after the complete absorption of the clot of blood. The evidence that they really arise from this cause is entirely complete; it might, indeed, be inferred from the facts which relate to the cases just pointed out, but if you are not able to follow every step of the reasoning, I would refer you to the work of Dr. Rochoux. The cyst in question occupied one of the usual seats of cerebral hæmorrhage.

In addition to these morbid changes, there were the traces of a large clot on the side of the brain, the conformation of which was obviously altered,

* This lecture did not, in the order of time, follow those which immediately precede it in the Examiner; but the obvious connexion of the subject with that of the last lecture, has induced us to adopt the present arrangement.

by a depression on the middle lobe of the left hemisphere, just behind the temporal muscle, quite unconnected with any alteration of the bone.

The membranes adhered very closely to the substance of the brain, and beneath them, was a partial softening of the medullary substance, which was of a light yellow or cream colour to the depth of about an inch, with complete destruction of the cortical substance in a space of two and a half or three inches square, that is in the whole extent of the depression, necessarily rendering that portion of the brain totally unfit for use. At the posterior part of this softened portion, was an imperfect cyst, more than an inch long, the walls of which were formed by a loose cellular substance, extending to the distance of from a quarter to a third of an inch. Near the centre of the same hemisphere, about an inch from the summit of the brain, was a third cyst, scarcely an inch long, of about half that breadth, and somewhat flattened. Its walls were formed by hard and yellow medullary substance,and it was filled with a transparent liquid. One fourth of the left hemisphere of the brain was, you thus see, destroyed; it was, besides, distorted and drawn back, to a degree that I never before witnessed; even the anterior portion of the brain was turned partially round and backward; this distortion may have interfered not a little, with the exercise of the functions of the brain, and was the necessary effect of cicatrization after a complete loss of cerebral substance.

From this disorganized condition of the brain, which rendered a large portion of it as useless as if it had been separated from the body, and caused the entire removal by absorption of another part, you may understand the cause of the complete paralysis of the right side of the body, and its necessary incurability, which I predicted. But, in addition to the immediate consequences of the apoplexy, other changes had taken place in the brain, not necessarily the result of hæmorrhage, as the recent active inflammation, from which the patient perished, which occurred from softening around the old cyst.

A symptom worth noting, was the loss of power of articulation, under which, you remember, the man laboured for a long period; his answers were confined to the word no, and were afterwards brief and confused. Now there was no lesion, except that caused by the contraction, in the anterior part of the brain, which, of course, disproves Bouillaud's assertion, that the vocal powers are connected with this portion—a point which had, indeed, been, previously, satisfactorily settled by the observations of Andral, and others. In this instance, the cortical substance of the brain was affected, although not in the anterior portion: the cortical substance, I have no doubt, presides over the functions of the intelligence and of the voice. The corpus striatum is supposed to preside over the faculties of motion of the upper extremity; and here, you see, the patient regained the power of walking, though not that of moving his arm. This, however, proves nothing; for it is a regular occurrence in hemiplegia, following apoplexy.

The therapeutics of this case are important; the impossibility of curing it is sufficiently evident, and, consequently, the necessity of confining your efforts, in similar cases, to such a plan of treatment as will palliate and improve the symptoms. Hence, too, you may doubt as to the propriety of addressing stimulating remedies to the brain and nervous system, as nux vomica, or its active principle, strychnia, to relieve a paralysis, dependent on destruction of the cerebral structure. These remedies were much in vogue at one of our institutions a few years ago, for the treatment of apoplectic hemiplegia. I witnessed most of the cases, and I never saw them produce decidedly good effects, although pushed so far, in some cases, as to produce convulsions. Many patients, afflicted with hemiplegia, in a degree recovered; but this occurred from the mere process of absorption of the clot, and not from the effect of the remedy. Indeed, I must candidly express my opinion against the usefulness of the remedy; and, as to its inconvenience, I am convinced that it often increases the activity of the circulation in the brain, surrounding the clot, from the over-stimulation of this organ. It is a valuable remedy in neuralgic paralysis, where there is functional disorder of the brain, or mere want of tone in the limb; but, where there is any considerable derangement of the cerebral structure, I am convinced that it is often a hurtful medicine, even when given in minute doses, and suspended as soon as its effects appear. I make these remarks upon the strychnia, because its use seemed indicated in one of these cases of paralysis; and although I anticipated but little effect from it, I consented to its administration; but it very soon became necessary to suspend it, from the increase of the difficulty of speech, and rigidity of the limbs. I am aware that many physicians of high judgment employ and recommend the strychnia; but my own observations, which were the more unbiassed, as they were made upon the practice of others, and not upon my own, have led me to a different conclusion. The true therapeutics in paralysis from apoplexy, consist first in subduing the inflammatory symptoms or the active congestion of the brain, by blood-letting, appropriate applications to the head, and purging; afterwards in waiting patiently and quietly, in keeping from the patient all causes of irritation, and in regulating his diet; and, after the clot has been removed, in addressing gentle stimulation to the paralysed part; or, what is better, in directing the patient to move it himself. Even this slight, and, as it were, natural mode of exciting the brain, may be attended with inconvenience. I lately directed a patient, in whom the paralysis was already of some months' standing, to move his arm every day by a powerful effort of will, and he went on until he succeeded in raising his hand to his head, but the brain became excited, he was stupid, and his speech thicker, and I was compelled to make him desist. Avoid, then, all causes of excitement, whether medicinal or other, in these cases of paralysis, which are either the mere effect of a considerable rupture of the fibres of the brain, or are connected with the subsequent inflammation, till very late in the treatment, and let it be confined to external stimulation.

The case which we have just been noticing, illustrates extremely well the advantages of a knowledge of pathological anatomy, in the study of diagnosis. We were able to define with exactness the morbid condition of the brain, as you may see from a report of the lecture which was published in the Examiner, which corresponds precisely with that which a post mortem examination has laid open. Now this verification, by means of examination after death of the lesions in a certain number of diseases, enables us to form a much more vivid and distinct picture of the state of the analogous cases. We conceive, as it were, in our mind, a well defined picture, and, by a sort of second sight, can discover most of the changes, which are, under ordinary circumstances, completely concealed. If we gain but little direct assistance in therapeutics, we obtain a sort of touchstone, by which we judge of the power of remedies, and thus acquire more accurate notions of the effect of medicinal agents ; we learn to discriminate between the natural course of a disease and the modifications impressed upon it by art. In itself, pathological anatomy is a mere instrument; but by its aid we are enabled to know positively a multitude of facts, which we can barely conjecture from the unaided study of symptoms. Now, I would impress upon you the necessity of not attaching an exaggerated importance to what is a mere means of investigation; you must never isolate the lesions of an organ from the symptoms which accompany them. I am the more earnest in insisting upon this matter, because you might imagine from the careful pathological investigations which I endeavour to make, that I value this sort of knowledge for its own sake ; this would be an error into which I should be loath to fall.

In concluding these lectures upon diseases of the brain, I have some few remarks to make upon certain functional affections of that organ, occurring during the course of various disorders of the body. These affections are very numerous, and often not a little puzzling in their character. For example, you no doubt supposed, when listening to the detail of symptoms denoting tubercular meningitis, that the features of the disease must be always clearly remarked, and yet there are affections that sometimes simulate it to a degree, that will embarrass a very experienced observer. It is no easy matter always to distinguish between diseases of the brain, itself, and those which are symptomatic of other affections. Now, this can only be done by becoming so familiar with these functional changes, that you may at once hold them up, as it were, in your mind's eye, and diagnosticate between them and the true cerebral diseases, by a rapid process of comparison, or, as it is sometimes termed, by way of exclusion. That is, you run over the list of these analagous disorders, and then rapidly leave them out of your calculations, because some essential symptoms may be wanting.

First, in fevers, intermittent and continued, particularly the latter, there occurs a train of cerebral symptoms, which are placed amongst the most important symptoms of these disorders. Continued fevers, in this section of country, are almost wholly the typhous and typhoid fevers, with the exception of the occasional occurrence of such as are styled bilious and ephemeral. In Paris, continued fever rarely takes any other type than the typhoid; while, in Ireland and Great Britain, it is generally the typhus. In both these fevers, the brain is affected at the commencement, but in a less manner than in meningitis, the early symptoms of both being headach and dizziness, with loss of strength. In the second stage, there is stupor, of great intensity in typhus, and of slighter in typhoid, often running into delirium. We have, at this time, also, in typhus, considerable alterations in the nervous system, indicated by spasms and subsultus, resembling those which occur in delirium tremens. The senses, also, are impaired in the second stage of both these affections, but more severely in typhus than in typhoid fever. In the last stage, we have coma, complete loss of the powers of intelligence and of motion, and very nearly complete suspension of the senses. Sometimes, we have violent, noisy delirium, which is to be looked upon as an irregular symptom, usually depending upon an accidental complication of meningitis with the fever; when this violent delirium occurs, it is always to be considered and treated as a secondary meningitis. The ordinary moderate cerebral symptoms, are, as it were, essential to the disease, and do not demand special interference, unless they should become intense, when they may be the immediate cause of death, and are to be treated as inflammations of the brain, by local depletion with cups and leeches, and by cold to the head, and the like. If this secondary meningitis of fever should occur very late in the disease, general bleeding is not often advisable. Treatment, although useless in slight cerebral symptoms, becomes essential, when they reach a high degree of activity. After coma supervenes, it is proper to abandon a depletory course, and the cold effusions, and you are now to resort to counter-irritants, sinapisms to the feet, blisters to the nucha,—remedies which are improper during the violent stage of the secondary meningitis. In the partial epidemic of typhus which occurred last winter, the fever was attended with more active cerebral symptoms than had previously shown themselves. I used local treatment, in nearly every case, with extreme advantage, and found that after removing the meningitis, the fever was almost free from danger.

In the intermittent and remittent fevers, the functions of the brain undergo alteration, although there is rarely active inflammation of the organ. There is less disturbance of the powers of motion than in the typhous and typhoid fevers, subsultus seldom occurring. The senses are not affected except in the height of the paroxysm, there is little ringing in the ears. These symptoms, however, are sometimes present in the malignant intermittents that we meet with in our hospitals, in the summer, occurring, principally, in sailors who have contracted the affection on the coast of North Carolina. In such cases, local depletion is not often advisable, for the cerebral symptoms are not confined to the paroxysm, nor do they resemble those of acute meningitis; they are rather loss of memory, sighing, and other signs of enfeebled nervous energy. They are best managed by large doses of quinine during the interval, and when in the paroxysm, with wine and volatile alkali. Upon these symptoms the danger of malignant intermittents chiefly depends. Of course this mode of treatment is not designed for those cases in which there are signs of more active vascular excitement, requiring the treatment of the acute cerebral symptoms of typhus. Your object must be to cure the intermittent.

In pneumonia, there is usually some slight disturbance of the brain, which, indeed, accompanies, in a greater or less degree, all febrile affections. Special treatment is required, only when there is either active delirium, or much stupor. When these exist, the case may be very readily mistaken by one not well accustomed to recognise pneumonia. In the cerebral complications of pneumonia, the peculiar flush of the face, the dyspnœa, and dilatation of the nostrils, serve to distinguish the nature of the affection, while, if there be meningitis of a primary character, it will be marked by the state of the eyes, frown of the forehead, and absence of the purple hue, and dark red flush. In the cerebral complication of pneumonia, a special treatment is occasionally demanded, consisting in purging, and antiphlogistics directed to the brain.

In inflammations of the serous membranes of the thorax or abdomen, the brain is rarely implicated, except to a slight extent, corresponding with the vascular excitement. The same may be remarked of inflammation of the mucous membranes; in affections of the bowels, the functions of the brain are not disordered, except in the last stage. If, however, the mucous membrane of a large extent of the alimentary canal be simultaneously attacked, then the brain sympathizes, and delirium very commonly ensues. In very severe epidemics of dysentery there is also extreme prostration of the nervous functions somewhat similar to what occurs in intermittent fevers.

The connection between functional disorder of the brain, and anemia, was alluded to in the last lecture, and illustrated very strikingly, by the history of a case which I then detailed. The sympathetic affection of the brain, in jaundice, is well known. We have a patient at this time, in the hospital, labouring under chronic gastritis and jaundice, in whom this cerebral alteration, depending on jaundice, is very manifest, and last year there were several marked cases of this kind. It is not of an inflammatory character; the symptoms being merely stupor and prostration, with subsultus, and particularly, loss of the memory. This set of symptoms indicates the connexion which exists between this affection and malignant intermittent and remittent fevers, and in both depends, in my opinion, upon the altered state of the blood which accompanies hepatic disease. Treatment is to be confined almost entirely to sinapisms and blisters, and occasionally some slight stimulants in addition to the general treatment for jaundice—cupping or other depletion should be rarely used. Dr. Marsh, an Irish physician of reputation, has also called the attention of the profession to the cerebral symptoms of jaundice, and agrees with me in opinion as to their great danger. Anemia, dependent on a vitiated condition of the liver, is to be treated by tonics, iron, porter, and a generous diet. But in many disordered conditions of the cerebral functions, the proper remedies are to be found amongst the narcotics and antispasmodics. The mild stimulant practice must, of course, be changed, if the patient get worse under it, or if you have any symptoms of more active inflammation of the brain. On the same principle is based the practice, recommended by Dr. Graves, for the sleeplessness and slight delirium in the latter stages of typhous fever, consisting in a combination of opium and tartar emetic. This is an excellent combination; the antimonial slightly nauseates, promotes gentle perspiration, and predisposes to sleep. The virtues of Dover's powder depend on the same union of opium with an analogous medicine, ipecacuanha, and, if the alimentary canal be in an irritated condition, this combination is to be preferred.

I have entered thus minutely into the detail of these functional cerebral symptoms, and into the points which distinguish them in different affections, because the symptoms, which are laid down in books, are nearly identical in all these affections. The order of symptoms, however, is very different and diagnosis becomes comparatively easy if we attend to their successive development.

---

### DELIRIUM TREMENS.

*Tuesday*, June 5th.—I shall, to-day, take up the subject of delirium tremens, postponing the consideration of diseases of the digestive organs, into which I had intended to enter, until they present themselves in greater numbers. Owing to the tardiness of the season, there is just now a singular paucity of them in our wards at the hospital, and I therefore defer the subject, until I can more amply illustrate it, which I will be able to do as the summer advances.

Delirium tremens is an affection which has special claims upon your attention, from the lamentable frequency of its occurrence, in our country. It is here, amongst the labouring classes, particularly the Irish, one of the most common of diseases, although in France and other continental countries of Europe, comparatively rare. During my residence in Paris, I did not see a single case of it: in the hospitals of that city, it is a disease that is never thought of, in patients who enter with cerebral symptoms, although with us, cases of delirium tremens are more numerous in our hospitals than those of all other cerebral diseases together.

I shall begin the subject, according to my usual practice, with the history of a case, illustrative of the affection. It is one, offering a mild type of it, and is, therefore, on many accounts, the better for study, as the symptoms are more characteristic of the disease in its early than in its latter stages, when they are more easily confounded with those of other forms of cerebral disturbance. The patient had been an inmate of the alms-house, previous to his entrance with mania a potu. He was an Irishman, about forty years of age, a clerk by occupation, reduced by habits of intemperance. He left the alms-house on the tenth of May, to look for work, and remained out for ten or twelve days, without drinking much, at the end of which time, he began to commit excesses, and, after a debauch of two or three days, slept out on a cold, rainy night, and was taken afterwards with a diarrhœa, which continued till yesterday. This diarrhœa he attributed, perhaps with reason, to the effects of cold, as he was pretty well ashamed of his drunkenness, and seemed anxious to magnify the symptoms, which were trifling, into a dysentery. In addition to the diarrhœa, he was taken, on Saturday last, with oppression across the breast, some cough, and also with vomiting, which arose, probably, from exposure, but may have been merely the termination of the debauch. He soon diminished the amount of spirits, which he was taking. In place of one or two quarts a day, he reduced his allowance to one or two glasses. On Sunday, the vomiting persisted, and so great was the irritability of his stomach, that he rejected even cold water; there was also purging. The nervous system now began to be affected, for he suffered from tremors, and the want of sleep on the previous night. These symptoms were as well known to the patient himself as they are to most habitual drunkards. At the entrance of the patient, we recognised a case of delirium tremens, which was well marked in all its features. There was nervous agitation, shown by the trembling of the hands and body, with obvious contraction of the pupils, which is nearly an infallible symptom in the beginning of delirium tremens; he had besides, restlessness, anxiety, and fear, disturbances of the mind, which are also strongly characteristic.

The man was placed yesterday under treatment, taking ℥ij. brandy with a small quantity of laudanum, to allay the irritability of his stomach. He slept eight hours last night, and his reason is now somewhat better. He is still labouring under a slight degree of mental hallucination, seeing various objects which he knows are not actually before him. For, in this stage of the affection, these patients are well aware, that the objects which they see have no real existence; when you talk to them on the subject, they will laugh, and tell you it is a mere notion from the horrors. In the more advanced state of the disorder, this distinction is lost, and they really believe the objects to exist, as the brain represents them.

I shall now proceed to detail to you the general symptoms, which characterise delirium tremens. The case of the man, whose history I have brought before you, may not present these symptoms in a regular series as you will have an opportunity of observing in other cases of the diseases where the symptoms are not checked by art. In the stage succeeding that in which he now is, you will find distinct hallucination of the senses, in which the objects presented to the mind, are nearly always of one kind, and give rise to fear, sometimes to the most intense terror. Thus, patients imagine that there are serpents crawling over the walls of their cells, that the house is falling in upon them, or is on fire, or that some person is about to discharge fire-arms upon them, from some real or imaginary aperture in the wall of the apartment. In the cells at the Pennsylvania Hospital, there is a hole in the wall, near the ceiling, for purposes of ventilation, and I there found a very common idea with patients was, to imagine that guns were pointed through this sort of loop-hole. The presence of tormenting devils is another vision not uncommon; in short, the patient is afflicted with the sight of almost every kind of terrible objects. This condition lasts for one or two days, at the end of which time the delirium loses its violent character, and is succeeded by incoherence, which indicates the approach of the third stage. There is none of this in the second stage, the patient being always able to talk in a continued and connected strain, while, in the third stage, his sentences are incomplete, and his tone is muttering—this latter symptom being very characteristic of the affection, and enabling us to pronounce at once as to its nature.

While these cerebral symptoms are advancing, another set is also taking place, in relation to the countenance and intellect. In the second stage, as you remarked yesterday, the expression of the face was that of fear, anxiety, and restlessness; still, when addressed, the patient could compose his countenance, and answer questions, if his memory was not too much taxed. But, in the third stage of the disease, the faculties of the memory are altogether lost, the patient being unable to utter a single sentence containing a rational answer. This fact is important in forming a diagnosis.

The next symptom is the condition of the pupils, which are almost always preternaturally contracted, during the second stage, and in the beginning of the third, and not dilating until effusion takes place: this contraction is sometimes extreme, the pupil not being larger than the point of a pin.

The state of the skin is next to be noted. There is often profuse sweating, particularly in the third stage. This you are to look upon as dangerous, from the weakness it induces, and as an indication of a very severe form of the disorder.

These are the chief symptoms connected with the state of the brain, except insomnium, which I mentioned, in describing the case of the man in the hospital. This is not, however, as has been asserted, the turning point of the disease; it is an important, but by no means, the chief or only sign.

Now let us examine into the condition of the alimentary functions. At the beginning of delirium tremens, they present but little variety. Anorexia is an almost invariable symptom: the patients have been drinking, until the powers of the stomach have become exhausted, and it then rejects every thing, even cold water, with disgust. This aversion to food is one of the symptoms by which you recognise the affection. The bowels are generally costive, but there is no regularity in their condition. There is thirst, from the fever, and irritation of the stomach, and increased by the restlessness of the patient.

These, then, are the chief symptoms which characterise delirium tremens. It might seem, at first sight, that they are so well marked, that there can be little danger of confounding them with those of any other disease. But delirium tremens often goes hand in hand with some other affection, and one of the two may be masked by the other. Hence, the difficulty of diagnosis. If there is delirium tremens alone, it is easy to recognise it; but if it occur along with inflammation of the brain, or with typhus fever, or in subjects who have been previously insane, you may be at a loss to distinguish it. It may also be complicated with congestion of the brain, and apoplectic and epileptic fits. How are you to diagnosticate it, under these various circumstances? You are, in the first place, distinctly to impress upon your minds the particular class of symptoms belonging to delirium tremens. The first of these is the tremor; then we have the intellectual disturbance, consisting in the fear of some supposed terrifying objects: this kind of mental disturbance does not occur with constancy, or even frequency, in any other disease of the brain; in inflammation of the membranes, there is much more commonly great irritability, and even disposition to commit actual violence. I saw a remarkable instance of this, last year. A soldier was brought into the hospital, who had attacked the recruiting sergeant and several of his comrades, it was supposed, from delirium tremens. However, I readily discovered his disease to be meningitis, from the character of the delirium, and the active circulation in the vessels of the head. He rapidly recovered under the antiphlogistic treatment already described. The next symptom, is the contracted condition of the pupils, which is a less peculiar sign. A third, is the power which the patient long retains of arresting his delirium, and directing his attention to other objects. The absence of all affection of the senses, other than hallucinations, is another point establishing the diagnosis: there is no paralysis, no subsultus—if these are present, you may suspect that you have inflammation of the brain, either alone, or complicated with delirium tremens. When meningitis and delirium tremens co-exist, some writers regard it merely as a modification of the latter affection, and lay great stress on its frequent occurrence during the fit of intoxication, before the alcoholic excitements have been suspended. The precise mode of commencement is, however, often difficult to determine; and I therefore attach much more importance to the symptoms, themselves, than to their origin.

The next point is, to distinguish between delirium tremens, and the disturbance of the brain, which occurs in typhus fever. If the latter disease prevail as an epidemic, the diagnosis will be troublesome: for the delirium of typhus, stupor, incoherence, &c., is very like the third stage of delirium tremens. There are, however, other symptoms of typhus, which may serve to establish the distinction; these are the effusion of blood throughout the cellular tissues of the body, the suffusion of the eyes, and the absence of the abundant sweats of delirium tremens. You will, however, occasionally meet with a combination of these affections, which occurs the more frequently because drunkards are especially liable to typhus. In these cases, the symptoms proper to delirium tremens subside as the stupor and muttering delirium of typhus become more prominent.

We now proceed to examine those cases which are complicated with organic or functional lesions of the brain, of a more chronic kind. These usually happen as follows:—A man, of a plethoric, full habit, drinks, until his intelligence becomes affected, and eyes suffused, and then falls into an apoplectic or epileptic fit. In persons predisposed to epilepsy, intemperance is apt to induce a fit of the disease, and the diagnosis remains exceedingly difficult, until you are able to obtain the previous history of the patient.

Those who are habitually of a very full and plethoric habit, are apt to fall into a kind of apoplectic fit from congestion of the brain. Hence, the intemperate, who are in easy circumstances, and are given to excesses in eating as well as drinking, are rarely attacked with delirium tremens without convulsions, and the disease is, in such persons, extremely dangerous. These cases are not to be managed according to the ordinary routine.

Although delirium tremens may complicate all possible diseases, I can by no means, however, admit intemperance to be the universal cause of disease among the poorer classes who fill our hospitals, that many persons suppose it to be. Although there was no delirium tremens in the hospitals of Paris, there was the same range of fatal diseases which we meet with here, independent, of course, of the complications resulting from the effects of drunkenness. The chief dangers of intemperance, consist in the demoralising tendency of this habit, and the inability which it induces to the performance of social and religious duties, which are surely quite enough to induce every one to use his exertions in checking the habits of using alcoholic drinks. But people die from its direct effects less frequently than is generally supposed. You must not, however, understand me to deny that diseases are rendered more fatal from previous intemperance; I am merely stating that its influence is often exaggerated; and, in science, I think we are bound to state those deductions which are founded on conscientious observation.

We now come to the causes of delirium tremens. The great cause, is a very simple one—an excessive indulgence in intoxicating drinks. The particular liquor, employed by patients whom we treat at the hospital, is, in nearly every case, whiskey. Brandy-drinkers are, usually, men in easy circumstances, who become habitual drunkards; they fare worse, in attacks of mania a potu, than the whiskey-drinkers, not that brandy is a more dangerous alcoholic combination, but that they who can afford to drink it, are able to procure it in larger quantities, and generally become confirmed topers, and are not obliged to pass through those periods of forced abstinence which occur with the poor, when their means of indulgence are exhausted. At the Pennsylvania Hospital, the mortality in delirium tremens is greater than at the Philadelphia, not from any superiority of treatment at the latter institution, but because the patients, who resort to the former, are persons in better circumstances of life, and have been indulging to a greater excess, and for a longer period.

The better and purer wines, the French, for example, never produce mania a potu. The same may be said of porter, ale, and the malt liquors, generally. The effect of the stronger wines cannot be well estimated, an excessive indulgence in them generally ending in the use of ardent spirits.

Delirium tremens may occur under one of several circumstances. First, a man may be in habits of daily drinking, but not to the extent of intoxication, say five or six glasses a day. He meets with an injury—breaks his leg, or the like—and is brought to the hospital for surgical treatment. He soon exhibits symptoms of pain and restlessness; and, the morning after the accident, you will find him in a state of anxiety and timidity, with tremors, and contraction of the pupils. These symptoms indicate the approach of an attack of delirium, and are often called "the horrors," from the dreadful feelings of the patient. They occur in persons in whom the habit of drinking has been suddenly interrupted. This condition is described by Dupuytren, and is by him supposed to depend simply upon the wound or other injury. You may, indeed, have these symptoms, occasionally, in persons of sober habits, and of a feeble, nervous temperament. It is, therefore, proper to be suspicious of this condition of things; but you must not be too hasty in setting it down as the incipient stage of delirium tremens. Soon after, however, you will have some symptoms, which will clear up the case, particularly the occurrence of delirium, of the character which I have described.

The second variety takes place, when alcoholic liquors are suddenly abandoned, that is, when the patient has suddenly abstained from an habitual and free use of them, to which he was accustomed without becoming absolutely intoxicated, or when he has suddenly ceased drinking after a debauch. In this case, delirium tremens usually shows itself on the third day, but, generally, under a mild form, and without much cerebral disturbance.

The third, and most common variety, is that which has presented itself in the patient whose case you have seen at the hospital, and which is always a bad form of the affection. A man drinks till his stomach fails, when he stops from pure inability to take in more, and begins to suffer from tremors. In this condition of the stomach, it may be that a smaller quantity of liquor is absorbed, and a less amount of stimulus has been directed to the brain. So that the disease may, perhaps, at last, arise from the subtraction of the accustomed excitement of the brain. These cases are apt to be extremely troublesome from a complication of gastritis with the cerebral disorder.

The fourth variety is common during the summer season. A man, accustomed to drink, works out in the sun, and the excessive heat developes inflammation of the brain, which becomes complicated with delirium tremens. In the summer of 1830, when the thermometer ranged above 90° in the middle of the days, during nearly the whole month of July, the effects of heat were so fatal, that persons of intemperate habits died in great numbers, either from sudden coma and convulsions, or, if they escaped the first effects of heat, from a complication of phrenitis and delirium tremens. This form of the disease is much more frequent in summer than at any other season of the year, and attended with much danger. This condition demands much discretion of management, being generally complicated with convulsions, and, altogether, a very unfavourable affection.

The duration of delirium tremens is a definite period, well established, from the non-interfering mode of treatment which has been sometimes adopted in its management—the patient is merely shut up and taken care of. I believe that this was the practice of the late Dr. Kuhn, and is nearly followed now in some institutions of Boston, where nothing is administered but a bitter infusion. We know, then, the duration of the second stage of mania a potu to be usually of three or four days, at the end of which period, the patient either dies or gets well. It may, however, run into a chronic state, or some other disease of the brain, or be followed by incurable mania.

The treatment of delirium tremens would seem to be, and is, in reality, very simple: if it be managed with judgment, the majority of patients get well. The treatment may be reduced to a few general rules, which are the following:

When a patient is brought to you, at the close of a debauch, which has been arrested by the failure of the powers of the stomach, the occurrence of a wound, or other cause, the indication is evidently to tranquillize the general nervous excitement, as well as allay the gastric irritability. The remedy most in use in this country, and in England, is opium. The practice of giving it in very large doses, originated in Philadelphia, and has long been in use at the Pennsylvania Hospital. Dr. Coates published a memoir on the subject, which attracted much attention. The practice recommended, was chiefly opiates, in very large doses, but with extreme care; the patient, if possible, was to be visited before each repetition of the dose. The practice passed to the hospital of the alms-house, and, when I was resident physician there, it was given in doses of three or four grains, carefully watching the effects. In bad cases, four grains were given every two hours, and continued till the patient was put to sleep, or until some symptom occurred to forbid it. This practice was abandoned, not because it was bad in itself, but because it was found that the same case called for a variety of remedies. The plan now is to regard opium as our sheet-anchor, in the treatment of delirium tremens, observing its effects, and abandoning it, or combining with it other remedies, when it does not succeed in relieving the symptoms. It may be given with safety, in doses of two grains, or forty drops of laudanum, every two hours, the patient being seen after every dose. In the ordinary delirium of mania a potu, I scarcely ever give it alone, combining with it other antispasmodics, or some form of alcohol. The good effects of this latter remedy, are well known to the vulgar, who are in the habit of tapering off, as they call it, by drinking less and less each day, when they wish to prevent the horrors. If, however, they have reached the extreme point of a debauch, they are not able to taper off in this way, the alcohol being rejected by the stomach; and it then becomes necessary to add laudanum to the spirits. I gave the patient, whose case was mentioned at the beginning of the lecture, twenty drops of laudanum, in a glass of brandy, and, by this means, was able to make him retain it, although he had before vomited immediately after taking it. This shows that by slightly varying your remedies, you may produce an effect, before unattainable. I have often reflected whether moral considerations ought to prevent the administration of alcoholic remedies; they have sometimes the disadvantage of keeping up the thirst for this kind of stimulus, longer than it would otherwise continue after a debauch, and, therefore, if other means are equally useful, the physician should abstain from their use. But, in a considerable proportion of cases of delirium tremens, particularly those of a slighter kind, they offer the readiest means of relieving the patient; and when you are satisfied that they are the best remedies for the particular case, you are bound in duty to your patients to resort to them.

If the irritability of the stomach be not great, other antispasmodics may be administered, as assafœtida, camphor, carbonate of ammonia, and Hoffman's anodyne. Of these, the assafœtida and anodyne are the best. Five grains of assafœtida, with a tea-spoonful of Hoffman's anodyne, and ten drops of laudanum, mixed up with the intervention of a little mucilage, given every hour, or every two hours, will often succeed in tranquillizing the patient; or, the laudanum may be given one hour, and the anodyne and assafœtida another. These remedies are directed to the nervous system; there are others to be addressed to the condition of the stomach. For this purpose, a very good one is capsicum, that I first saw employed by Dr. Hewson, of this city. He tried it in a patient, in whom other remedies had failed, a young man, of good fortune, who finally destroyed himself by intemperance. He gave the capsicum in the dose of a grain, in pill, every hour, until he relieved the irritability of the stomach, and was able to act on the nervous system, with opium and assafœtida. Other remedies are employed in New England, as the infusion of wormwood, used in the penitentiary at Boston, which, by restoring the tone of the stomach, acts on the nervous system.

Emetics were recommended, some years ago, by Dr. Klapp, of this city, in the treatment of delirium tremens, and were tried in the alms-house, though afterwards abandoned, for a return to other remedies, the efficacy of which is consecrated by long use. Emetics may do good in stout, robust patients, at the very beginning of the affection, when a dose of ipecacuanha will sometimes break up the train of morbid associations, and arrest the disease. They often quickly relieve in the horrors or the initial stage of the disease. But if the stomach be irritable, and the patient prostrated, emetics, particularly tartar emetic, are manifestly inadmissible. I saw them tried, a year ago, under these circumstances, and the patient was lost. I have tried them myself, and, under favorable circumstances, have succeeded in checking the disease. In bad cases, and where there is prostration, they can do no good, and should not be given hastily.

[The remainder of this lecture will be given in the next number.]

